# Loss of Akt1 or Akt2 delays mammary tumor onset and suppresses tumor growth rate in MTB-IGFIR transgenic mice

**DOI:** 10.1186/1471-2407-13-375

**Published:** 2013-08-07

**Authors:** Katrina L Watson, Roger A Moorehead

**Affiliations:** 1 Department of Biomedical Sciences, Ontario Veterinary College, University of Guelph, Guelph, ON N1G2W1, Canada

## Abstract

**Background:**

Akt is a serine/threonine kinase that mediates signaling downstream of tyrosine kinase receptors like the type I insulin-like growth factor receptor (IGF-IR). In fact, we have previously shown that mammary tumors induced by elevated expression of the IGF-IR are associated with hyperactivation of Akt. However, there are three mammalian isoforms of Akt (Akt1, Akt2 and Akt3) and these isoforms regulate distinct physiologic properties within cells. In this manuscript, the impact of disrupting Akt1 or Akt2 in mammary tumors induced by IGF-IR overexpression were examined to determine whether specific Akt isoforms regulate different aspects of mammary tumorigenesis.

**Methods:**

Akt1 and Akt2 levels were stably ablated in mammary tumors of MTB-IGFIR transgenic mice by crossing MTB-IGFIR transgenic mice with either Akt1^−/−^ or Akt2^−/−^ mice. Tumor onset, growth rate, and metastasis were determined.

**Results:**

Ablation of Akt1 or Akt2 significantly delayed tumor onset and tumor growth rate but did not significantly alter lung metastasis. Despite the absence of Akt1 or Akt2, mammary tumors that developed in the MTB-IGFIR mice maintained detectable levels of phosphorylated Akt. Disruption of Akt1 or Akt2 did not affect cell morphology or the expression of luminal or basal cytokeratins in mammary tumors.

**Conclusions:**

Although loss of Akt1 or Akt2 significantly inhibited mammary tumor onset and growth rates the effects were less dramatic than anticipated. Despite the complete loss of Akt1 or Akt2, the level of total phosphorylated Akt remained largely unaffected in the mammary tumors suggesting that loss of one Akt isoform is compensated by enhanced activation of the remaining Akt isoforms. These findings indicate that therapeutic strategies targeting the activation of individual Akt isoforms will prove less effective than simultaneously inhibiting the activity of all three Akt isoforms for the treatment of breast cancer.

## Background

The insulin-like growth factor (IGF) family has been implicated in a number of human cancers including breast cancer [1-5]. In particular, the type I IGF receptor or IGF-IR has been found to be expressed at high levels in 39-93% of human breast cancers [6-9]. Originally, the IGF-IR was associated with luminal breast cancer however more recent studies have found the IGF-IR in all breast cancer subtypes [9-14]. Two different transgenic mouse models have also shown the importance of IGF-IR in mammary tumorigenesis. In a study by Carboni et al. [15] expression of a constitutively active IGF-IR in mammary epithelial cells induced mammary tumor development. The other transgenic model was developed in our lab and this model overexpressed the wild type IGF-IR in mammary epithelial cells in a doxycycline inducible manner [16]. Overexpression of wild type IGF-IR also resulted in the formation of mammary tumors.

One important signalling molecule downstream of the IGF-IR is Akt. Akt is a serine-threonine kinase that lies downstream of PI3K signaling [17-21]. There are 3 Akt isoforms in mammals and each is transcribed from separate genes [22-25]. Based on genetic ablation of each isoform in mice it appears that Akt1 regulates cell survival and/or proliferation based on the observation that Akt1^−/−^ mice display increased perinatal lethality and are smaller in size [26]. Akt2 appears to regulate glucose homeostasis and Akt2^−/−^ mice develop insulin-resistant diabetes while Akt3 appears important in the brain as Akt3^−/−^ have impaired brain development [27,28]. The function of Akt isoforms with respect to breast cancer has been investigated in cell lines and transgenic models of mammary tumorigenesis. In MDA-MB-231 human breast cancer cells, which express low levels of activated Akt, the overexpression of constitutively active forms of Akt1 or Akt2 inhibited cell proliferation and migration with little effect on apoptosis [29]. In a non-transformed human mammary epithelial cell line (MCF-10A) genetically altered to express high levels of IGF-IR, Akt1 downregulation reduced proliferation and enhanced migration while Akt2 downregulation reduced proliferation but had not effect on migration [30].

The function of Akt isoforms has been studied in two transgenic mammary tumor models, MMTV-neu and MMTV-PyMT. MMTV-neu transgenic mice constitutively overexpress erbB2 in mouse mammary epithelial cells using the mouse mammary tumor virus (MMTV) promoter. MMTV-PyMT transgenic mice express the polyoma virus middle T antigen in mammary epithelial cells using the MMTV promoter. In both models overexpression of constitutively active Akt1 was shown to delay mammary tumor formation but had no effect on metastasis while the overexpression of activated Akt2 did not affect tumor latency but did enhance tumor metastasis [31]. In addition, ablation of Akt1 in MMTV-neu or MMTV-PyMT transgenics inhibited tumor formation while the ablation of Akt2 accelerated tumor formation [32]. In addition, loss of Akt1 in MMTV-neu tumors enhanced their invasiveness.

In our MTB-IGFIR transgenic mice, we found that Akt1 or Akt2 ablation significantly increased in tumor latency and decreased tumor growth rate. Moreover, loss of Akt1 or Akt2 did not alter tumor histology or cytokeratin expression.

## Methods

### Ethics

Animals were housed and cared for following guidelines established by the Central Animal Facility at the University of Guelph and the guidelines established by the Canadian Council of Animal Care. This study was approved by the Animal Care Committee at the University of Guelph.

### Mice

MTB-IGFIR transgenic mice were generated in our lab and have been previously described [16]. Akt1^−/−^ and Akt2^−/−^ mice were purchased from Jackson Laboratories (Bar Harbor, ME). Since the Akt1^−/−^ and Akt2^−/−^ mice were in a C57BL/6 background, these mice were backcrossed 7 times with FVB mice to generate Akt1^−/−^ and Akt2^−/−^ mice in the same genetic background as our MTB-IGFIR transgenic mice. The MTB-IGFIR transgenic mice were then mated with either Akt1^−/−^ or Akt2^−/−^ mice until the appropriate genotypes were obtained. MTB-IGFIR, MTB-IGFIR/Akt1^−/−^ and MTB-IGFIR/Akt2^−/−^ mice were administered chow supplemented with 2 g of doxycycline per gram of chow beginning when the mice were 21 days of age.

### Tumor measurement and collection

All mice were monitored 2 times per week by palpating the mammary glands. Once a palpable mammary tumor was identified the age of the mouse was recorded and tumor growth was monitored using digital calipers. The formula, *volume = length × width*^*2*^*/2* was used for estimating tumor volume. Tumor growth rate was calculated using the formula for specific growth rate (SGR); [SGR = ln (V_2_/V_1_)/(t_2_-t_1_)] [33]. Once the mammary tumors reached either 17 mm in diameter or 10% of the mouse’s body weight, the mice were euthanized and the mammary tumors were collected. Each mammary tumor was collected and divided for fixation in formalin, cryopreservation in OCT and flash frozen.

### Western blotting

Western blotting was performed as described in Jones et al. [16]. All antibodies were obtained from Cell Signalling Technologies (Beverly, MA) except for the IGF-IR antibody which was obtained from R&D Systems (Minneapolis, MN) and the β-actin antibody which was obtained from Sigma (Oakville, ON). All antibodies were used at a 1:1000 dilution except for β-actin which was used at a 1:5,000 dilution. Appropriate secondary antibodies were obtained from Cell Signalling Technologies (Beverly, MA) and used at a dilution of 1:2,000. Images were captured on a FluorChem 9900 gel documentation system (Alpha Innotech, San Leandro, CA) and quantification of western blots was performed using AlphaEase software (Alpha Innotech, San Leandro, CA).

### Histology and immunohistochemistry

Mammary tumors and lungs were collected and processed as previously described [16,34]. Immunohistochemistry was performed as previously described [16]. Primary antibodies were used at a dilution of 1:200 and were obtained from the following sources, anti-Ki67, anti-cytokeratin 5 and anti-cytokeratin 14 (Abcam, Cambridge, MA), anti-cytokeratin 18 (Research Diagnostics Inc, Flanders, NJ), and anti-cytokeratin 8 (Fitzgerald Industries International Inc, Concord, MA). Primary antibodies were detected using a 1:200 dilution of the appropriate secondary antibody and Sigma Fast 3,3′-diaminobenzidine tablets (Sigma, St. Louis, MO). Ki67 immunohistochemistry was quantified using Positive Pixel Count software v9 (Aperio, Vista, CA) following slide scanning on a ScanScope CS slide scanner (Aperio, Vista, CA).

### Statistics

An ANOVA followed by a Duncan’s post-hoc test was used to determine statistical significance since the groups had an unequal sample size and the Duncan’s test adjusts for sample size. Values with p<0.05 were considered statistically significant.

## Results

**Figure 1:**
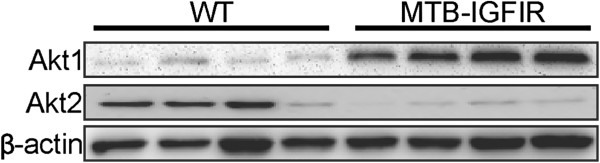
**The levels of Akt1 and Akt2.** Western blot for Akt1 and Akt2 in wild type (WT) mammary tissues from four different mice and mammary tumors from four different MTB-IGFIR transgenic mice. Akt3 was evaluated but could not be detected in any of the samples. The WT tissue was taken from adult mice that had not been exposed to doxycycline. β-actin served as a loading control.

Previous characterization of the MTB-IGFIR transgenic mice showed that mammary tumors expressing high levels of IGF-IR also contained high levels of phosphorylated Akt [16]. However, it was not determined which of the Akt isoforms were important in the MTB-IGFIR mammary tumors. Therefore, as an initial experiment, the levels of the three Akt isoforms were evaluated in normal, wild type (WT) mammary tissue and in mammary tumors of MTB-IGFIR transgenic mice. Evaluation of Akt1, Akt2 and Akt3 revealed that Akt1 and Akt2 can be detected in both WT mammary tissue and mammary tumors (Figure 1) while Akt3 could not be detected in either WT mammary tissue or mammary tumors.

**Figure 2:**
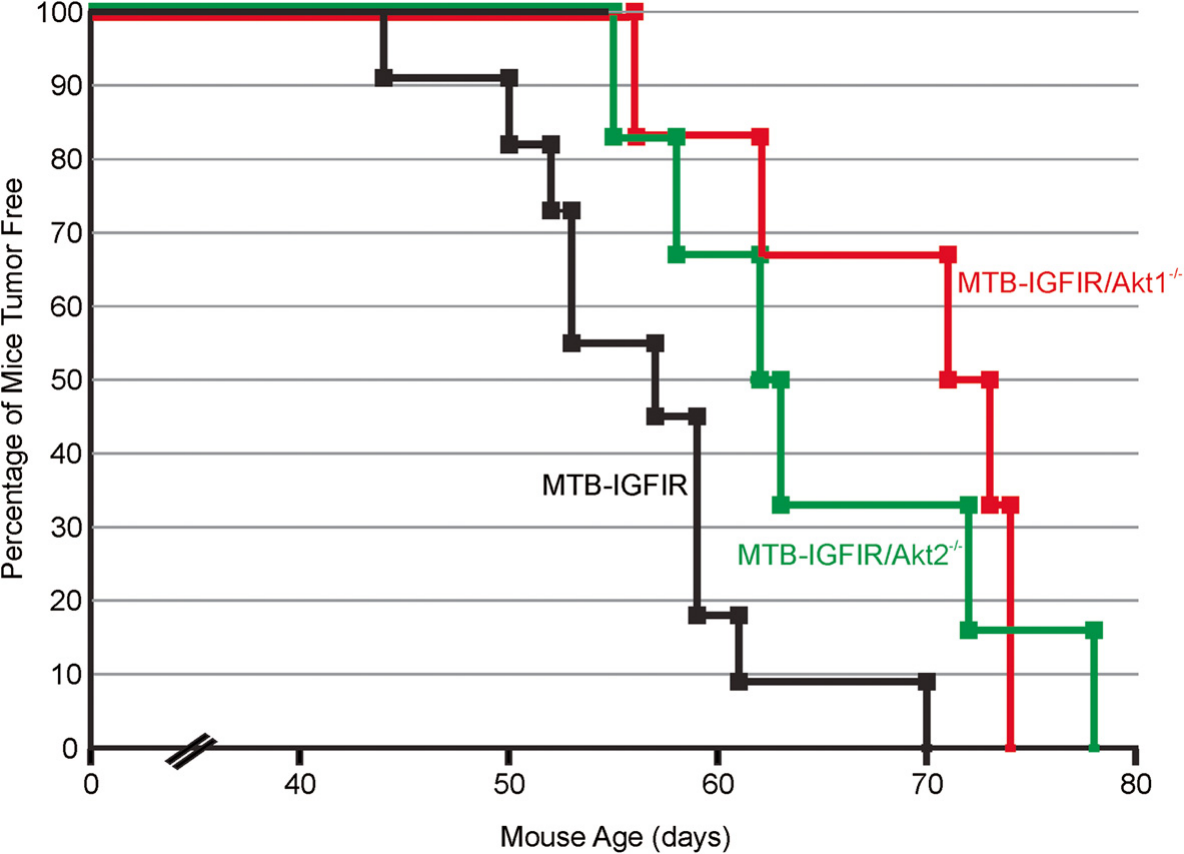
**Mammary tumor onset in MTB-IGFIR, MTB-IGFIR/Akt1**^**-/- **^**and MTB-IGFIR/Akt2**^**-/- **^**mice.** The graph shows mammary tumor onset in MTB-IGFIR/Akt1^-/-^ mice (red line; n=6), MTB-IGFIR/Akt2^-/-^ mice (green line; n=6) and MTB-IGFIR mice (black line; n=11). The same group of MTB-IGFIR mice were used for both comparisons.

Since Ak1 and Akt2 were consistently expressed in the mammary tumors, MTB-IGFIR mice were crossed with Akt1^−/−^ and Akt2^−/−^ mice to determine the roles of Akt1 and Akt2 in mammary tumorigenesis. To minimize any effects that mouse strain may play on mammary tumorigenesis, the Akt1^−/−^ and Akt2^−/−^ mice were backcrossed 7 times into a FVB/N background so that all mice were in a FVB/N background (MTB-IGFIR transgenic mice were created and maintained in a FVB/N background). MTB-IGFIR/Akt1^−/−^, MTB-IGFIR/Akt2^−/−^, and MTB-IGFIR mice were fed food supplemented with 2 g of doxycycline per kilogram of rodent chow beginning when the mice were 21 days of age. Mice overexpressing IGF-IR and containing Akt1 and Akt2 developed tumors at approximately 56.5 days of age or 35.5 days after the initiation of doxycycline supplemented food. This onset is similar to what we previously published for these mice [16]. In the MTB-IGFIR/Akt1^−/−^ mice, tumor onset was 47.3 ± 3.1 days after supplying the mice with doxycycline supplemented food (Table 1). This tumor onset was approximately 12 days longer than the MTB-IGFIR mice and this difference was statistically significant. In the MTB-IGFIR/Akt2^−/−^ mice tumor onset was 43.6 ± 3.6 days after the addition of doxycycline to the food and this delay in tumor onset was also statistically different from the MTB-IGFIR mice (Table 1). Graphs showing the tumor onset are presented in Figure 2. There were no obvious differences in the number of tumors that developed in each animal or the number of animals with lung metastasis (Table 1).

**Table 1:** Tumor characterization

**Genotype**	**# of mice**	**Tumor onset (days)**	**Specific growth rate**	**# of tumors at time of death**	**# of mice with lung metastases**
MTB-IGFIR	11	35.5 ± 2.0	0.14 ± 0.02	3.2 ± 0.2	2/11
MTB-IGFIR/Akt1^−/−^	6	47.3 ± 3.1*	0.07 ± 0.01*	2.8 ± 0.8	1/6
MTB-IGFIR/Akt2^−/−^	6	43.6 ± 3.6*	0.09 ± 0.02*	3.5 ± 0.6	1/6

*significantly different from WT as determined by a Duncan’s test using WT, Akt1^−/−^ and Akt2^−/−^ mice.

**Figure 3:**
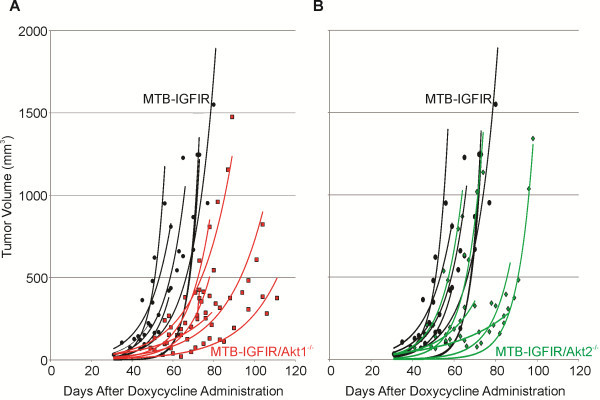
**Mammary tumor volume in MTB-IGFIR, MTB-IGFIR/Akt1**^**-/- **^**and MTB-IGFIR/Akt2**^**-/- **^**mice.** Graphs showing tumor volumes in **(A)** MTB-IGFIR/Akt1^−/−^ mice (red squares, red lines; n=6) compared to MTB-IGFIR mice (black circles, black lines; n=11) and **(B)** MTB-IGFIR/Akt2^−/−^ mice (green diamonds, green lines; n=6) compared to MTB-IGFIR mice (black circles, black lines; n=11). Tumor volumes for each mouse were plotted as a scatter plot and the best fit, exponential line was drawn. The same group of MTB-IGFIR mice were used for both comparisons.

Tumor growth rates were also evaluated using the formula for Specific Growth Rate (SGR) [33]. It was observed that mammary tumors in MTB-IGFIR/Akt1^−/−^ mice had SGRs that were approximately half that of the MTB-IGFIR mice. This difference in growth rate was significant. The mammary tumors in MTB-IGFIR/Akt2^−/−^ mice had growth rates that were approximately 36% slower than MTB-IGFIR mice and this difference was also statistically significant. Figure 3 shows a scatter plot with best fit exponential tumor growth curves to illustrate the differences in mammary tumor growth rates. As indicated in these plots there was some variability regarding growth rates of mammary tumors in MTB-IGFIR/Akt1^−/−^ and MTB-IGFIR/Akt2^−/−^ mice, however, the majority of the tumors grew at a slower rate than the mammary tumors of MTB-IGFIR mice.

**Figure 4:**
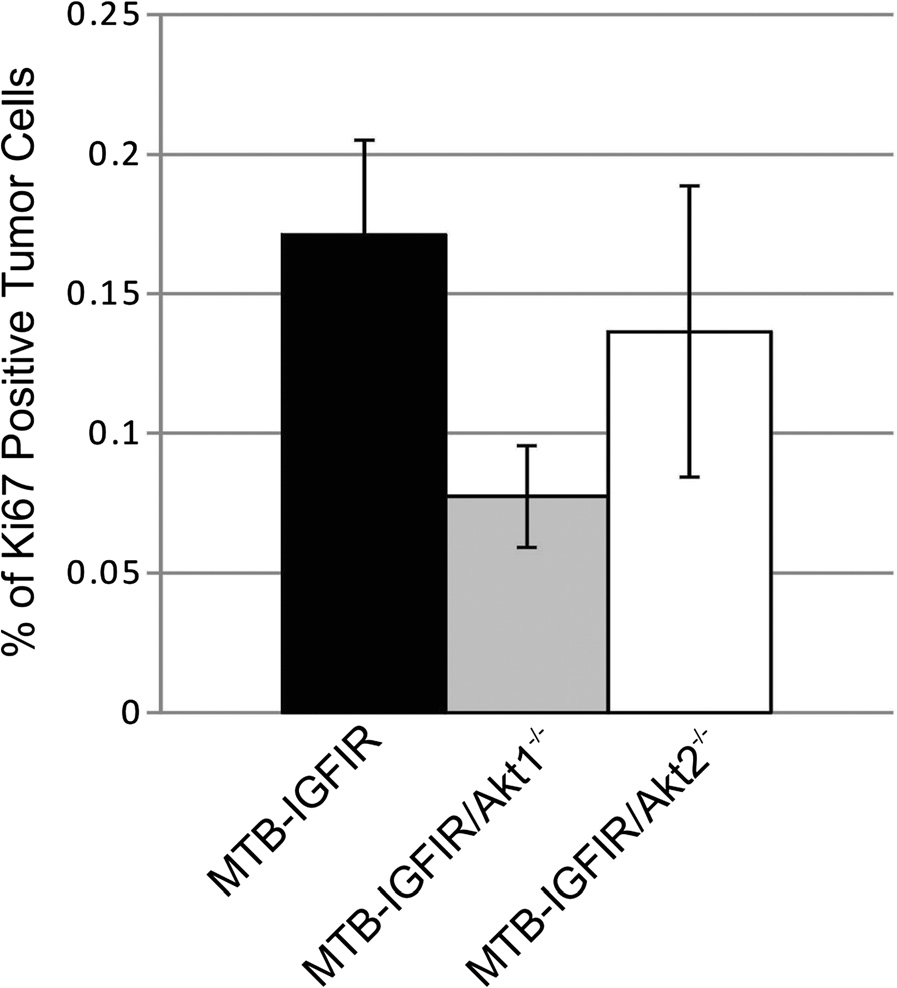
**Mammary tumor proliferation in MTB-IGFIR, MTB-IGFIR/Akt1**^**-/- **^**and MTB-IGFIR/Akt2**^**-/- **^**mice.** The mitotic index of mammary tumors from MTB-IGFIR mice (black bar; n=11), MTB-IGFIR/Akt1^−/−^ mice (grey bar; n=6) and MTB-IGFIR/Akt2^−/−^ mice (white bar; n=6) was determined using Ki67 immunohistochemistry. The bars represent the mean and standard error of the percentage of tumor cells positive for Ki67 staining.

To determine whether tumor proliferation was affected in either the MTB-IGFIR/Akt1^−/−^ or MTB-IGFIR/Akt2^−/−^ mice Ki67 immunohistochemistry was performed. It was observed that tumor cell proliferation in the MTB-IGFIR/Akt1^−/−^ tumors was reduced approximately 55% compared to MTB-IGFIR tumors while proliferation rates in the MTB-IGFIR/Akt2^−/−^ tumors were reduced approximately 20% (Figure 4). The data was however quite variable and thus neither result were statistically significant.

**Figure 5:**
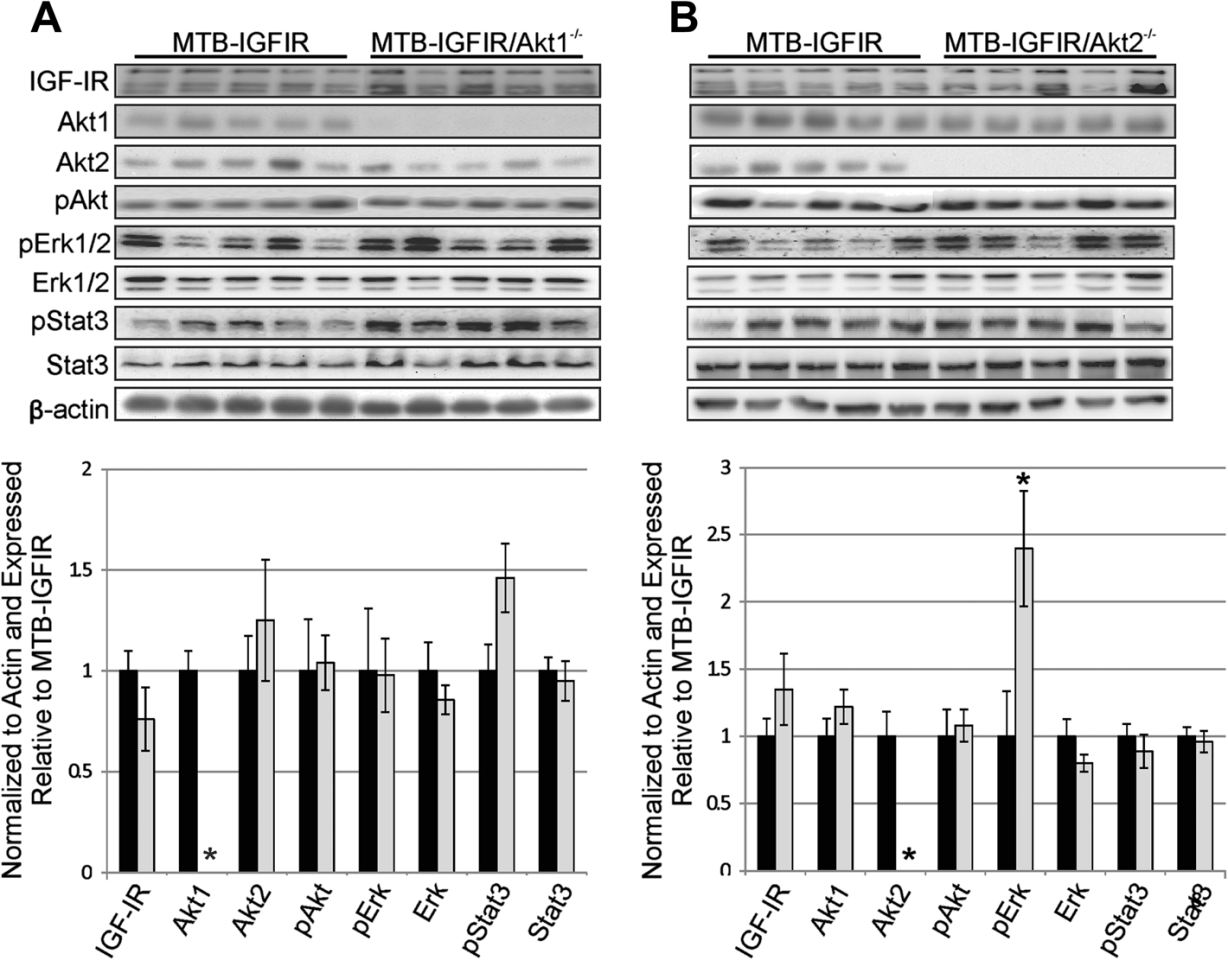
**The levels of signaling proteins in mammary tumors from MTB-IGFIR, MTB-IGFIR/Akt1**^**-/- **^**and MTB-IGFIR/Akt2**^**-/- **^**mice.** Western blot for IGF-IR, Akt1, Akt2, phosphorylated Akt (pAkt; Ser^473^), phosphorylated Erk (pErk), Erk, phosphorylated Stat3 and Stat3 in **(A)** mammary tumors from MTB-IGFIR transgenic mice compared to MTB-IGFIR/Akt1^−/−^ mice or **(B)** mammary tumors from MTB-IGFIR transgenic mice compared to MTB-IGFIR/Akt2^−/−^ mice. Each lane represents tissue from a different animal and the relative abundance of each protein is presented in the bar graph below the western. *p<0.05 as determined by a Student’s *T*-test.

To confirm protein levels in the mammary tumors, western blotting was performed for IGF-IR, Akt1, Akt2, phosphorylated Akt (pAkt), Erk1/2, phosphorylated Erk1/2 (pErk1/2), Stat3 and phosphorylated Stat3 (pStat3). As shown in Figure 5, mammary tumors with normal levels of Akt1 and Akt2 had similar levels of the IGF-IR as mammary tumors null for Akt1 or null for Akt2 (Figure 5A,B). The levels of Akt1 were undetectable in MTB-IGFIR/Akt1^−/−^ mammary tumors while the levels of Akt2 were similar in MTB-IGFIR mammary tumors, and mammary tumors from MTB-IGFIR/Akt1^−/−^ mice. Similarly, the levels of Akt2 were undetectable in MTB-IGFIR/Akt2^−/−^ mice while Akt1 levels were similar in MTB-IGFIR mammary tumors and mammary tumors from MTB-IGFIR/Akt2^−/−^ mice. The levels of Akt3 could be detected at very low but similar levels in the mammary tumors from three different genotypes (data not shown). Despite the loss of Akt1 or Akt2, the mammary tumors from MTB-IGFIR/Akt1^−/−^ and MTB-IGFIR/Akt2^−/−^ mice had levels of phosphorylated Akt similar to the mammary tumors that developed in MTB-IGFIR mice. The levels of some of the signalling molecules downstream of phosphorylated Akt were also found at similar levels in all genotypes except for higher levels of phosphorylated Erk1/2 associated with MTB-IGFIR/Akt2^−/−^ tumors (Figure 5A,B).

**Figure 6:**
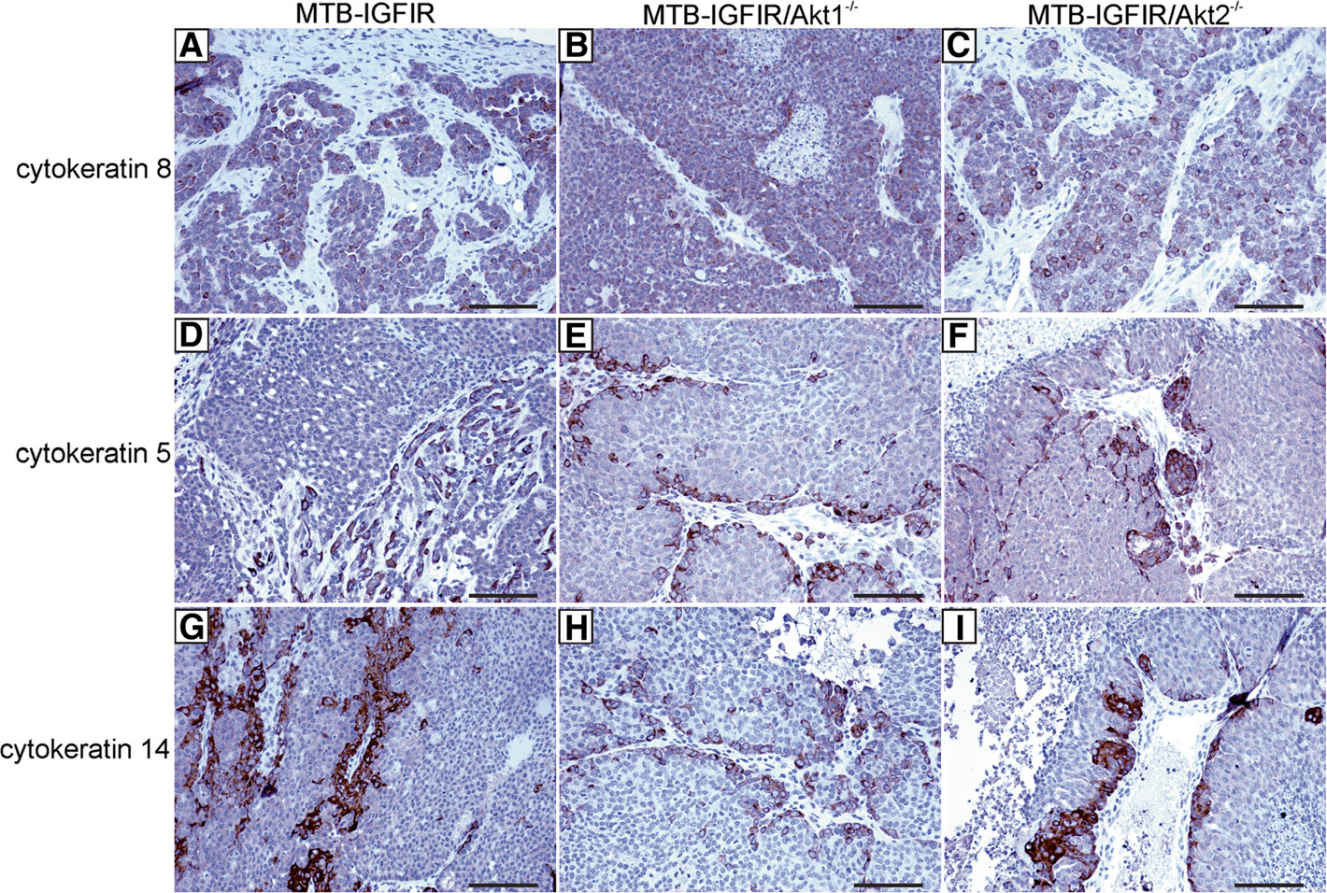
**Cytokeratin expression in mammary tumors from MTB-IGFIR, MTB-IGFIR/Akt1**^**-/- **^**and MTB-IGFIR/Akt2**^**-/- **^**mice.** Immunohistochemistry for cytokeratin 8 **(A-C)**, cytokeratin 5 **(D-F)** or cytokeratin 14 **(G-I)** in mammary tumors from MTB-IGFIR mice **(A,D,G)**, MTB-IGFIR/Akt1^−/−^ mice **(B,E,H)** or MTB-IGFIR/Akt2^−/−^ mice **(C,F,I)**. The brown color indicates the presence of each protein while the nuclei of the cells are blue due to hematoxylin staining. Scale bars, 100 μm.

Since Akt1 and Akt2 have been implicated in epithelial to mesenchymal transition in some mammary models [30,35,36], immunohistochemical analysis of cytokeratins 5, 8, 14 and 18 was performed to determine whether the type of mammary tumors that developed in MTB-IGFIR/Akt1^−/−^ or MTB-IGFIR/Akt2^−/−^ mice differed from the tumors that developed in MTB-IGFIR mice. The majority of the mammary tumors in each genotype contained cells that expressed moderate or high levels of cytokeratin 8 (Figure 6A-C) and/or 18 (data not shown) indicating these cells were luminal in nature. However, some tumors contained clusters of cytokeratin 5 and 14 positive cells and these clusters were independent of Akt genotype (Figure 6D-I). This mixture of tumor with luminal and basal characteristics was observed in the original characterization of the MTB-IGFIR mammary tumors [16].

## Discussion

Since Akt is one of the most hyperactive kinases in cancer [17] it is an attractive therapeutic target. However, there are three Akt isoforms and our understanding about the functions of the individual Akt isoforms in normal and cancerous tissue is incomplete. In our study we found that loss of either Akt1 or Akt2 produced a small but significant delay in tumor onset and a small but significant decrease in tumor growth rate.

Delayed mammary tumor onset and reduced tumor growth rate observed in the MTB-IGFIR/Akt1^−/−^ mice was consistent with the findings of Maroulakou et al. [32] which showed that loss of Akt1 significantly delayed tumor onset and reduced tumor growth in MMTV-neu and MMTV-PyMT transgenic mice. However, loss of Akt1 in the MMTV-neu and MMTV-PyMT transgenic mice had a much more dramatic impact on tumor onset than that observed in the MTB-IGFIR transgenic mice. Maroulakou et al. [32] also observed that loss of Akt2 enhanced mammary tumorigenesis, a result not observed in the MTB-IGFIR/Akt2^−/−^ mice. In addition, Maroulakou et al. [32] observed that loss of Akt1 reduced lung metastasis in MMTV-PyMT but not MMTV-neu transgenic mice. Neither loss of Akt1 nor Akt2 significantly affected metastasis in the MTB-IGFIR transgenic mice however it should be noted that decreased metastatic rate in the MTB-IGFIR transgenic mice may be difficult to detect as these tumors inherently have a relatively low metastatic rate.

There are several differences in the studies involving the MMTV-neu or MMTV-PyMT transgenics and our MTB-IGFIR transgenics. First, the transgene used to produce the mammary tumors was different. In our case, the IGF-IR drove mammary tumorigenesis while in the study by Maroulakou et al. [32], either ErbB2 or the polyoma virus middle T antigen were used. Although IGF-IR and ErbB2 are both tyrosine kinase receptors that initiate similar signalling cascades, tumors induced by these two transgenes are distinct. Using gene expression profiles, we previously showed that our MTB-IGFIR mammary tumors did not cluster with the MMTV-neu or MMTV-PyMT mammary tumors [37]. The MTB-IGFIR mammary tumors clustered most closely with human basal tumors [37] while the MMTV-neu mammary tumors have been reported to cluster more closely with human luminal breast cancers [38]. The MMTV-PyMT transgenics, like the MMTV-neu transgenics, clustered most closely with human luminal breast cancers [38].

Another potential significant difference was the strain of the mice. The study by Maroulakou et al. [32] did not specify the mouse strains used however Akt1^−/−^ and Akt2^−/−^ mice are typically in a C57BL/6 background while MMTV-neu and MMTV-PyMT transgenic mice are typically in a FVB background. Therefore, it is unclear whether this study was performed on a mixed strain background. In our study, Akt1^−/−^ and Akt2^−/−^ mice were backcrossed 7 times into a FVB background and mated with MTB-IGFIR transgenic mice which are on a FVB background. Therefore, our studies were performed on an essentially pure genetic background. It should be noted that we also attempted to backcross our MTB-IGFIR transgenic mice into a C57BL/6 background however even on the first backcross, these mice failed to form mammary tumors (unpublished observations).

In our study, alternative signaling pathways appeared to compensate for the loss of Akt1 or Akt2. This was evident by the fact that Akt phosphorylation did not decrease in mice lacking either Akt1 or Akt2. Since the total level of Akt phosphorylation did not diminish despite the absence of Akt1 or Akt2, it appears that the tumor cells compensated by increasing the phosphorylation of the remaining Akt isoforms. An increase in Erk phosphorylation was also observed in the MTB-IGFIR/Akt2^−/−^ tumors compared to MTB-IGFIR tumors. Erk1/2 signaling promotes survival and may have contributed to tumor cell survival in the absence of Akt2. Compensation between these pathways has been observed in mammary tumors in previously published studies [39,40]. It is also possible that signaling pathways not evaluated in this study may have compensated for the absence of Akt1 or Akt2. The paper by Maroulakou et al. [32] did not evaluate the activation of intracellular signaling molecules in their tumors so comparisons to our study cannot be made.

Despite previous reports implicating Akt in EMT [30,35,36] we found no evidence that loss of Akt1 or Akt2 affected tumor cell morphology in the MTB-IGFIR transgenic mice. However, since EMT was not a focus of this study this property was not extensively examined in the current study. EMT is a process whereby epithelial cells convert to a mesenchymal morphology and express genes typically associated with mesenchymal cells (reviewed in [41-44]). EMT is thought to increase the invasiveness of breast cancers [45-49].

## Conclusion

Although loss of Akt1 or Akt2 significantly inhibited mammary tumor onset and growth rates these effects were very modest. It is probable that the loss of Akt1 or Akt2 was compensated for by the remaining Akt isoforms and/or alternative signaling pathways in the tumor cells. Compensation has important clinical implications in that tumors are unlikely to significantly respond to therapeutic agents targeting individual signaling pathways or individual protein isoforms within signaling pathways. Future studies using Akt inhibitors for the treatment of breast cancer should employ Akt inhibitors that target both Akt1 and Akt2 in combination with inhibitors that target additional signaling pathways such as the MAPK signaling pathway.

## Abbreviations

Akt1−/−: Knockout mice null for Akt1; Akt2−/−: Knockout mice null for Akt2; Dox: Doxycycline; EMT: Epithelial-to-mesenchymal transition; Erk: Extracellular signal-regulated kinase; FITC: Fluorescein isothiocynate; HER2: Human epidermal growth factor receptor 2; IGF: Insulin-like growth factor; IGF-IR: Type I insulin-like growth factor receptor; MMTV: Mouse mammary tumor virus; MTB-IGFIR: Transgenic mice expressing human type I insulin-like growth factor receptor in a doxycycline-inducible manner; MTB-IGFIR/Akt1−/−: Transgenic mice expressing human type I insulin-like growth factor receptor in a doxycycline-inducible manner that are also null for Akt1; MTB-IGFIR/Akt2−/−: Transgenic mice expressing human type I insulin-like growth factor receptor in a doxycycline-inducible manner that are also null for Akt2; MMTV-neu: Transgenic mice expressing neu/ErbB2 in mammary epithelial cells; MMTV-PyMT: Transgenic mice expressing polyoma virus middle T antigen in mammary epithelial cells; pAkt: Phosphorylated Akt; PBS: Phosphate buffered saline; PBS-T: Phosphate buffered saline with 0.2% triton-×100; pErk: Phosphorylated Erk; PI3K: Phosphoinositide 3-kinase; pIGF-IR: Phosphorylated IGF-IR; pStat: Phosphorylated Stat; RM11A: Mammary tumor cell line established from a mammary tumor that developed in a MTB-IGFIR mouse; RNAi: Ribonucleic acid interference; SGR: Specific growth rate; Stat: Signal transducer and activator of transcription; WT: Wild type

## Competing interests

Both authors declare that they have no competing interests.

## Authors’ contributions

KLW performed all of the *in vivo* work and experiments using tissue derived from the animal models. RAM ran the project and wrote the manuscript. Both authors read and approved the final manuscript.

## Pre-publication history

The pre-publication history for this paper can be accessed here:

http://www.biomedcentral.com/1471-2407/13/375/prepub
